# Interpersonal reactivity index adaptation among expectant seroconcordant couples with HIV in Zambézia Province, Mozambique

**DOI:** 10.1186/s40359-020-00442-0

**Published:** 2020-08-28

**Authors:** Daniel E. Sack, Michael B. Frisby, Matthew A. Diemer, Caroline De Schacht, Erin Graves, Aaron M. Kipp, Almiro Emílio, Ariano Matino, Ezequiel Barreto, Sara Van Rompaey, Kenneth A. Wallston, Carolyn M. Audet

**Affiliations:** 1 Vanderbilt Institute for Global Health, Vanderbilt University Medical Center, 2525 West End Ave, Suite 750, Nashville, TN 37203 USA; 2 School of Education, University of Michigan, Ann Arbor, MI USA; 3 Friends in Global Health, Maputo, Mozambique; 4 Friends in Global Health, Quelimane, Mozambique; 5 Vanderbilt Institute for Medicine and Public Health, Nashville, TN USA

**Keywords:** Empathy, HIV/AIDS, Dyadic analysis, Scale validation

## Abstract

**Background:**

The ability to understand another’s emotions and act appropriately, empathy, is an important mediator of relationship function and health intervention fidelity. We adapted the Interpersonal Reactivity Index (IRI) – an empathy scale – among seroconcordant expectant couples with HIV in the Homens para Saúde Mais (HoPS+) trial – a cluster randomized controlled trial assessing couple-based versus individual treatment on viral suppression – in Zambézia Province, Mozambique.

**Methods:**

Using baseline data from 1332 HoPS+ trial participants (666 couples), an exploratory factor analysis assessed culturally relevant questions from the IRI. Because empathy is interdependent among couples, we validated the results of the exploratory factor analysis using a dyadic confirmatory factor analysis (CFA) with dyadic measurement invariance testing. Finally, we assessed the relationship between scores on our final scale and basic demographic characteristics (sex, age, education, and depression) using t-tests.

**Results:**

We found two subscales: 1) a seven-item cognitive empathy subscale (Cronbach’s alpha 0.78) and 2) a six-item affective empathy subscale (Cronbach’s alpha 0.73). The dyadic CFA found acceptable model fit and metric invariance across partners (Comparative Fit Index (CFI) = 0.914, Tucker Lewis Index = 0.904, Root Mean Squared Error of Approximation = 0.056, ΔCFI = 0.011). We observed higher cognitive (*p*: 0.012) and affective (*p*: 0.049) empathy among males and higher cognitive (*p*: 0.031) and affective (*p*: 0.030) empathy among younger participants. More educated participants had higher affective empathy (*p*: 0.017) and depressed participants had higher cognitive empathy (*p*: < 0.001). This two-subscale, 13-item version of the IRI measures cognitive and affective empathy in HoPS+ trial participants and adults while accounting for the interdependent nature of empathy within partner dyads.

**Conclusions:**

This scale will allow us to assess the interplay between empathy and other psychometric constructs (stigma, social support, etc.) in the HoPS+ trial and how each relates to retention in HIV, adherence to treatment, and prevention of maternal to child HIV transmission. Furthermore, this scale can be adapted for other sub-Saharan African populations, which will allow researchers to better assess HIV-related intervention efficacy.

**Trial registration:**

This study is within the context of the HoPS+ trial, registered at ClinicalTrials.gov as number NCT03149237. Registered May 11, 2017.

## Background

As of 2017, there were 36.9 million people living with HIV (PLWH) globally, 19.6 million of whom lived within Eastern and Southern sub-Saharan Africa (SSA) [1]. In Zambézia Province, Mozambique, HIV prevalence is estimated to be 15% within the general population, with higher estimated rates in pregnant women [2]. Given the well-documented association between higher maternal HIV viral load and higher likelihood of infant HIV diagnosis [3], understanding the role of empathy and partner empathy - within the medical, social, economic, and cultural setting of rural Mozambique – on retention in care, adherence to treatment, and maternal-to-child transmission among PLWH is essential to decreasing HIV/AIDS-related morbidity and mortality in Mozambique and globally. With the increased focus on couples-based interventions to improve HIV outcomes in SSA, [4–10] understanding the interpersonal skills of each member within a couple, such as empathy, may be key mediators of the couples-based intervention efficacy. For example, partners who are better able to understand each other’s perspective may be better able to support them in adherence to antiretroviral medication regimes. However, to our knowledge, an empathy scale has never been validated in or adapted anywhere in SSA, which limits our ability to measure the impact of empathy on retention in HIV care in this setting.

### Introduction to empathy, partner empathy, and the interpersonal reactivity index

Empathy, the ability to understand another’s emotions and act accordingly, has a cognitive domain — the ability to understand the experiences of others — and an affective domain — emotional responses to others’ experiences [11–13]. Empathy is considered essential to understanding the mental states and experiences of others, a key component of social behavior that allows individuals to function within groups [12, 13]. Specific to this study, empathy is recognized as a key mediator of health intervention fidelity and, within the context of HIV, higher physician empathy is associated with improved patient outcomes [14–17]. It stands to reason that partner empathy would increase emotional support, treatment adherence, retention in care, and relationship quality – all of which contribute to reducing mother-to-child transmission of HIV.

Recently, there has been increased interest in examining empathy among partners, given that partner interdependence within a dyad results in survey item correlation among partners [18–20]. These new methods recognize how one partner’s affective empathy influences the other partner’s cognitive empathy and vice versa [20]. They also show that one partner’s empathy is related to supportive behaviors directed towards the other partner [21]. These between-partner relationships demand a methodological approach that recognize subject interdependence (i.e., does not assume subject independence).

The Interpersonal Reactivity Index (IRI) was developed to assess the distinct cognitive and affective empathy domains [11]. Péloquin and Lafontaine [19] developed and used a modified version of the IRI to assess empathy among couples [19]. Levesque used a dyadic model to further validate the updated IRI scale [19, 20]. We could not, however, find evidence of IRI validation among couples outside of North America. This may limit its assessment of empathy in individuals in other regions, such as within SSA, in individuals with lower levels of education, and within the context of a dyadic relationship outside of North America. This has important implications for interventions targeted to improve health outcomes, including interventions for people living with HIV in SSA.

### The current study

This study uses baseline data, specifically demographic data and the IRI, from an ongoing cluster randomized controlled trial — Homens para Saúde Mais (HoPS+) [Men for Health Plus] — that assesses the impact of involving HIV-positive male partners in routine prenatal care for women living with HIV [5]. The HoPS+ trial represents a unique opportunity to take the first step in adapting a measure that will allow researchers to assess empathy, as well as changes associated with behavioral interventions, in Mozambique and, with subsequent studies, in SSA.

The purpose of this study was to adapt the IRI among study participants in the ongoing HoPS+ study through an exploratory factor analysis, dyadic confirmatory factor analysis, and dyadic measurement invariance testing. We used a dyadic approach to account for how each partner’s perceived empathy impacts the other partner’s perceived empathy. This informs understanding of how supportive behaviors, shaped by between-partner interdependence, may augment engagement with and outcomes from HoPS+ and other behavioral interventions. Further, we assess correlations between the adapted IRI measure and related demographic and psychological (e.g., depression) factors, to provide further convergent and divergent validity evidence for the adapted measure. These results will help us evaluate the effect of our intervention on male and female empathy in males and pregnant women living with HIV in Mozambique and SSA and lay the groundwork for future assessments of dyadic constructs, including empathy, in SSA.

## Methods

### Participant selection

The HoPS+ study protocol is described in detail elsewhere [5]. Briefly, trained local study personnel collected baseline age, sex, education, marital status, occupation, IRI, and Patient Health Questionaire-9 data using a REDCap® (Research Electronic Data Capture) survey administered to pregnant women living with HIV and their seroconcordant male partner from 24 clinic sites in Zambézia Province [5, 22].

This analysis included the first 666 couples, or 1332 individual participants, from all HoPS+ study sites beginning on the date of study initiation (November 16th, 2017) through June 13th, 2019, when data were downloaded from our REDCap® database [22]. Our final study population, after excluding 147 participants (42 complete couples and 51 additional individuals) for missing data, included 1185 individuals (567 complete couples and 51 additional individuals) from 24 sites (Table 1). This included 595 (50.2%) females and 590 (49.8%) males with a median age of 25 (Interquartile Range (IQR) 21–30) and 5 years (IQR 2–7) of education. The most common occupation was farming (46.9%). Three districts, Pebane (30%), Inhassunge (16.5%), and Namacurra (19.3%), were overrepresented in the validation sample as compared with the excluded sampled population (18.4, 1.4, and 8.8%% respectively, *p*-value < 0.001), likely because when we randomly selected HoPS+ sites, more were located in these three districts than the other two. Furthermore, because of their size, recruitment was initially faster than the more remote, smaller districts. Mungia (43.1%) and Chuabo (37.6%) were the most commonly spoken languages, based on the most popular language(s) in each study district.

**Table 1:** Patient Demographic Information

Sample	Validation (*n* = 1185)	Excluded (*n* = 147)	χ^2^ Test
	*Frequency (%) or Median (IQR)*	*Frequency (%) or Median (IQR)*	*p-value*
Baseline Age	25 (21, 30)	23 (20, 28)	
*Sex*			0.727
Female	595 (50.2)	71 (48.3)	
Male	590 (49.8)	76 (51.7)	
*Relationship Status*			0.001*
Single	488 (41.2)	62 (42.2)	
Married	249 (21)	49 (33.3)	
Domestic Partnership	448 (37.8)	36 (24.5)	
*Highest Education*			0.001*
Total Years	5 (2, 7)	5 (3, 7)	
None	182 (15.4)	11 (7.5)	
Primary (≤ 7 yrs)	789 (66.6)	99 (67.3)	
Secondary (> 7 yrs)	214 (18.1)	37 (25.1)	
*Occupation*			0.901
Farmer	556 (46.9)	72 (49)	
Domestic Worker	322 (27.2)	38 (25.9)	
Other	303 (25.6)	37 (25.1)	
*District*			< 0.001*
Pebane	355 (30)	27 (18.4)	
Inhassunge	196 (16.5)	2 (1.4)	
Gilé	134 (11.3)	18 (12.2)	
Quelimane	21 (1.8)	3 (2)	
Mocubela	156 (13.2)	48 (32.7)	
Namacurra	229 (19.3)	13 (8.8)	
Maganja da Costa	94 (7.9)	36 (24.5)	
*Predicted Language*			< 0.001*
Muniga	511 (43.1)	75 (51)	
Chuabo	446 (37.6)	18 (12.2)	
Lomué	134 (11.3)	18 (12.2)	
Nharringa	94 (7.9)	36 (24.5)	

*IQR* interquartile range

* indicates statistical significance at *α* = 0.05

### Setting

Zambézia Province, located in north-central Mozambique, is home to approximately 4.4 million people from five primary ethnic groups (Chuabo, Macua-Lomwe, Manhaua, Merenge, and Senas) who speak at least four languages in our study area [5]. It has some of the poorest health and development indicators in Mozambique. Mozambique’s literacy rate is 47%; only 28% of women are literate (vs. 60% of men) and these numbers are lower in rural communities [23]. The majority of inhabitants are subsistence farmers [23]. Nationwide, 40% of the population live in poverty, but 80% of those poor live in rural areas like Zambézia [24]. In addition to these contextual conditions, the HIV prevalence in the province is estimated to be 15%, one of the highest in the country [2].

### Scale translation and adaptation

This is a novel setting for employing the IRI, and this population presented unique challenges in study implementation. Measures were translated (and back translated to confirm meaning was maintained) from English to Portuguese (a shared language among translators, and a commonly spoken language in Mozambique) and then from Portuguese to Muniga, Chuabo, Lomue, and Nharringa. At least seven trilingual interpreters carefully reviewed each study question and made modifications relevant to the local sociocultural and linguistic context. Specific phrases, including feeling “touched” were not translated verbatim, but were replaced with similar, locally relevant concepts. The final measure was subsequently field tested at each study site before enrolling participants. During interviewer-assisted survey implementation (due to low levels of literacy among participants), statements and response categories were read aloud in each participant’s preferred language on enrollment. Responses were captured by the study counselor. Twenty-four trained counselors fluent in the local language and Portuguese were trained to capture participant responses over two 5-day training sessions. All counselors were supervised in the field by a study manager, who provided regular booster trainings to ensure consistency of survey delivery.

### Instruments

#### Interpersonal reactivity index

The IRI consists of four empathy domains with seven questions each (28 questions total) [11]. The fantasy scale (FS) assesses one’s ability to place oneself in fictional situations; the perspective-taking scale (PT) reflects one’s ability to understand another person’s point of view; the empathic concern scale (EC) measures one’s ability to have caring feelings towards another individual; and the personal distress scale (PD) characterizes an individual’s own negative feelings when witnessing adverse events in others [11]. The fantasy and perspective taking scales constitute the cognitive component of empathy, while the empathic concern and personal distress scales constitute the affective component of empathy [11]. More recent research, albeit conducted in North America, further supports that distinct cognitive and affective empathy domains undergird the IRI scale [25–29]. This includes the development of two-factor empathy scales [25, 28] and imaging and molecular research that suggest distinct, but interrelated, cognitive and affective neural circuitry [27, 29].

Each item is scored on a 5-point Likert-like scale ranging from “Does not describe me well” (0) to “Describes me very well” (4). Although the original IRI contained nine reverse scored items, in the above-described scale adaptation, all questions were positively phrased and scored to avoid confusion during translation and survey administration as well as to improve response accuracy. Previous studies report Cronbach’s alpha values for IRI subscales from 0.70–0.83 and correlation coefficients of 0.01–0.37 between subscales [11, 30–32].

#### Patient health Questionaire-9

The Patient Health Questionaire-9 (PHQ-9) measures the nine attributes that characterize major depressive disorder [33]. Participants rate each attribute from ‘Not at all’ (0) to ‘Nearly every day’ (3) and were considered depressed if they scored 10 or greater. The PHQ-9 has been validated to screen PLWH in SSA for depressive symptoms [34–36] and has been used to measure depressive symptoms in Mozambique [37]. Participants who disclosed suicidal ideation (item 9 on the PHQ-9) were immediately assessed by our trained counselors and referred to the psychologist based at each site for additional psychological services.

### Missing data

We excluded participants with missing IRI data on more than eight questions (~ 30% of answers), an average of more than two questions per subscale (*n* = 147; 11%). We believed that these criteria excluded potentially biased data from interactions among interviewer-interviewee pairs who had difficulty administering or understanding the survey, given the low levels of education among our participants. Those missing more than one PHQ-9 item (*n* = 124; 10.5%) were excluded from our analysis as described above. We used a stricter threshold for the PHQ-9 because it has previously been used in Mozambique and because it was a shorter measure.

Missing IRI and PHQ-9 data for the participants with eight or fewer missing IRI items (*n* = 276; 23.3%) and one or fewer missing PHQ-9 items (*n* = 112; 9.4%) were imputed over 10 data sets using non-missing empathy survey questions with the multivariate imputation by chained equations (mice) package version 3.4.0 [38]. We used a chi-squared test to assess the differences between included and excluded participants by sex, marital status, district, highest education, occupation, and predicted language (based on the most frequently spoken language in each district because we did not collect individual level data on language).

Included (*n* = 1185) and excluded (*n* = 147) participants did not statistically differ by age or occupation (Table 1). However, there were statistically significant differences by relationship status – a higher percentage of included participants self-identified as in a domestic partnership (37.8 to 24.5%) and fewer self-identified as married (21 to 33.3%). Included and excluded participants also had different levels of education and were differentially representative of districts (*p* < 0.001) and predicted languages (*p* < 0.001) (Table 1).

### Data preparation

IRI questions were translated into several new languages and administered to participants in Zambézia, who were markedly different from the college-educated and junior high school students in previous IRI validations [19, 20, 30–32]. Although it has been used in South Africa as a composite 28-item scale or in its 4-subscale form [39, 40], to our knowledge the IRI has never been validated in or adapted to SSA. Given the new context, language translation, and rephrasing of negatively worded items, we did not feel comfortable making the a priori configural assumptions necessary to start with a confirmatory factor analysis of the IRI. We therefore hypothesized that the factor structure might differ from the previously identified four-factor structure [11]. We randomly split, without replacement, the full sample of 666 couples into two groups to identify (in the first phase) and then confirm, via confirmatory factor analysis (in the second phase) the factor structure of the IRI in discrete dyadic samples. Data were treated as ordinal in both EFA and CFA validation analyses. All data cleaning and analysis was conducted in *R*, version 3.5.1 (R Foundation for Statistical Computing) (2018-07-02) [41].

### Exploratory factor analysis

The exploratory factor analysis (EFA) included 400 individuals (200 dyads split for the analysis). After re-examining the question translations for this analysis, we removed questions number 4 and 9 from the pool of items subjected to analyses, due to discrepancies after translation that changed the meaning of the questions (Supplementary Table 1). We performed maximum-likelihood exploratory factor analyses on the polychoric correlation matrices of two, three, and four-factor solutions consistent with the affective and cognitive attributes of empathy and IRI for couples scale (two factor-model suggested by extant theory) [19, 25–29], the results of a parallel analysis on the imputed datasets (three factor-model suggested by parallel analyses) [42, 43], and previous versions of the IRI (four factors in the initial validation) [11]. We used an oblique promax rotation and selected items for a particular factor if the loading was greater than 0.40 and unique to one factor (i.e., the question did not load greater than 0.40 for another factor; if so, it was discarded). We assessed internal factor consistency with Cronbach’s alpha to compare our results with previous IRI psychometric evidence.

### Dyadic confirmatory factor analysis and measurement invariance

**Fig. 1:**
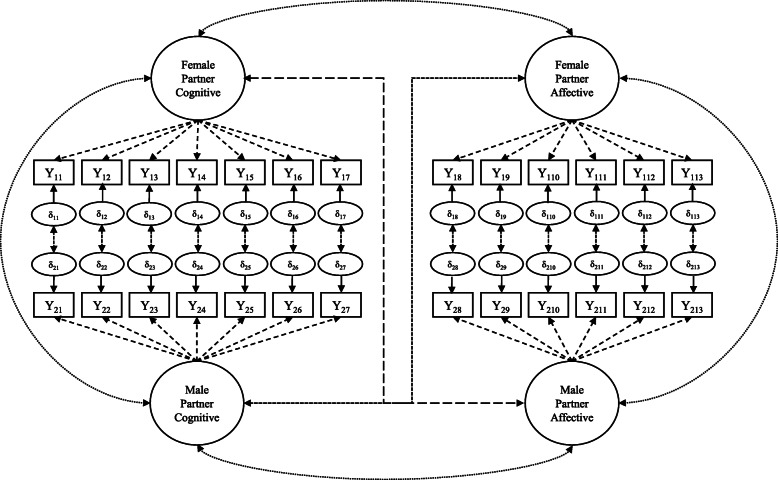
A Conceptual Diagram of the Dyadic Empathy Confirmatory Factor Analysis. Cognitive and Affective dimensions of empathy are uniquely modeled for male and female partners. Dashed lines from each empathy construct to the corresponding items (boxes) indicate the relation between the construct and the items used to model it. Circles pointing to items represent each item’s error term. Covariances, represented by the curved and dashed lines between the constructs, capture the relationships between partners’ dimensions of empathy. Double-headed arrows between error terms capture the interdependence between partners’ responses to survey items

We used a dyadic confirmatory factor analysis (CFA), which included 466 dyads distinct from those analyzed in the EFA, to capture the interdependence between partner responses to items on the IRI (Fig. 1). This perspective presupposes that the latent construct being modeled achieves measurement invariance [44]. This means that the latent construct — empathy in this case — means the same thing and is measured the same way across partners. Thus, measurement invariance needs to be tested as part of the dyadic model validation. We examined three progressively restrictive levels of measurement invariance — configural invariance, metric invariance, and scalar invariance — using latent variable analysis (lavaan version 0.6–3) [45]. Statistical power for invariance testing may be “reasonable” with a sample of 200 or more dyads and “adequate” with 400 dyads [46]. Robust absolute and incremental fit indices are reported using standard benchmarks [47–50].

*Configural* invariance assesses statistical equivalence of factor loading patterns across both partners. Configural invariance provides evidence that, when underlying latent constructs are measured between partners, items are organized in a similar fashion (i.e., the same set of items measure the same latent constructs for both partners). Constructs exhibiting *metric* invariance confirm statistically equivalent factor loading values across latent constructs and actors. Metric invariance provides evidence that items are similarly related to the underlying constructs across both actors. Finally, *scalar* invariance confirms that items are expected to have the same value across actors when the corresponding latent construct equals zero. In other words, the scaling of items is equivalent across partners. Scalar invariance indicates whether the amount of variation in each group is equivalent, and that each groups’ mean differences are interpretable [51, 52]. Power analyses for invariance testing suggests the use of changes in alternative fit indices (ΔAFI). Because the sensitivity of model fit indices may vary by sample size and model specification, simulations on sample sizes larger than 300 suggests evaluating model differences via changes in the confirmatory fit index (ΔCFI) between .002 and .01, and changes in the root mean squared error of approximation (ΔRMSEA) between .007 and .015. ΔRMSEA has been shown to be more sensitive than ΔCFI [46, 53]. Thus, due to the uncertainty of the novel context and language translation, we chose to use the more conservative options of ΔCFI > .002, and did not consider ΔRMSEA (though we do report it) for evaluating measurement invariance.

We report adjusted (robust) and unadjusted fit indices for each configural, metric, and scalar invariance model (see Table 4). Robust fit indices reflect modifications to traditional fit indices to account for failure to meet distributional assumptions, such as non-normality [47, 48]. These adjustments to absolute (Root Mean Squared Error of Approximation - RMSEA) and incremental (Comparative Fit Index (CFI) & Tucker Lewis Index (TLI)) fit indices make use of the Satorra-Bentler scaling constant to modify the equations of the naïve fit estimates [54]. Simulation studies have found these adjustments to be robust across a variety of unmet distributional assumptions and attenuate the overestimation of fit quality.

### Demographic comparisons of cognitive and affective empathy

To further probe the construct validity of the resulting factor model, we assessed inter-scale correlation, correlation between subscales and the continuous PHQ-9 scores, and the relationship between each scale and sex, age, education, and depression. We used unpaired t-tests to assess the relationships between each subscale and the dichotomous measure of sex, age (> 26 years-old), education (> 7 years), and depression (> 9 PHQ-9 score is depressed). We hypothesized that participants who were older (> 26) would have lower empathy scores given their exposure to the war of independence and/or civil war and its sequelae which devastated Zambézia Province [55, 56]; that women would have higher empathy scores consistent with previous validations [30–32]; that those with more depressive symptoms – due to a higher degree of internalization of difficult emotional situations [57–60] – and that participants with higher levels of education would have higher levels of empathy, which has been observed in other settings [55]. We then assessed mean empathy subscale scores by district and predicted language with a one-way analysis of variance.

## Results

### Exploratory factor analysis

All the exploratory factor analyses (343 individuals) resulted in factor structures that mixed items from the four IRI subscales, and, to a lesser extent, between the cognitive and affective attributes of empathy (Table 2). In the absence of a theoretical framework to explain mixing of cognitive and affective items, we selected the two-factor solution (distinct cognitive and affective empathy factors) to minimize these discrepancies. This aligns with more recently developed empathy scales and the number of factors on the IRI for couples scale, which allows for improved interpretability of the two-factor solution instead of the three- or four-factor solutions, which had subscale mixing that limited interpretability [25, 28]. Across ten imputed datasets, factor correlations ranged from 0.56 to 0.59. We then removed cognitive questions that loaded on the predominantly affective scale and vice versa to maintain the integrity of previous empathy scale frameworks [11, 30–32].

**Table 2:** Interpersonal Reactivity Index Exploratory Factor Analysis Question Loading Across Ten Multiply Imputed Iterations (*n* = 343)

Question (subscale)	Factor 1	Factor 2
1) I imagine and dream, with some regularity, about things that might happen to me. (FS)	0.24–0.39	0.14–0.36
2) I often have feelings of affection and concern for people less happy than me. (EC)	*0.34–0.44*	0.17–0.29
3) I can see things from “another person's” point of view. (PT-)	0.11–0.18	**0.46–0.59**
**5) I really get involved with the feelings of the characters in a movie. (FS)**	**0.44–0.54**	0.04–0.24
**6) In emergency situations, I feel afraid and ill- disposed. (PD)**	0.03–0.12	**0.40–0.51**
7) I’m not normally objective when I watch a movie or game, and I often get completely caught up in it. (FS-)	0.23–0.36	*0.23–0.42*
8) I try to look at everybody’s side of a disagreement before I make a decision. (PT)	*0.36–0.45*	−0.01-0.16
**10) Sometimes I feel helpless when I am in the midst of a very emotional situation. (PD)**	0.11–0.23	*0.36–0.50*
11) Sometimes, to try to understand my friends better, I imagine how things seem from their perspective. (PT)	*0.36–0.43*	0.14–0.27
12) It’s a common for me to become heavily involved in a good book or movie. (FS-)	−0.27--0.18	**0.52–0.60**
**13) When I see someone get hurt, I usually don’t stay calm. (PD-)**	−0.15--0.09	**0.65–0.72**
**14) The misfortunes of other people usually disturb me much. (EC-)**	−0.31--0.21	**0.61–0.75**
15) If I’m sure I’m right about something, I spend time listening to other people’s arguments. (PT-)	0.19–0.24	0.20–0.34
**16) After seeing a play or movie, I feel like I’m one of the characters. (FS)**	**0.54–0.56**	− 0.07-0.02
**17) Being in an emotional and tense situation scares me. (PD)**	−0.03--0.01	**0.43–0.50**
**18) When I see someone being treated unfairly, I feel much pity for them. (EC-)**	−0.18--0.14	**0.54–0.62**
19) I tend to be ineffective in dealing with emergencies. (PD-)	*0.37–0.43*	0.13–0.26
20) I am often very touched by things that I see happen. (EC)	*0.39–0.46*	0.08–0.21
**21) I believe there are two sides to every question and I usually look at both. (PT)**	**0.51–0.58**	−0.07-0.03
22) I would describe myself as a very kind person. (EC)	**0.49–0.61**	−0.25--0.18
**23) When I watch a good movie, I can easily put myself in the place of the main character. (FS)**	**0.67–0.78**	−0.23−− 0.14
24) I tend to lose control during emergencies. (PD)	0.15–0.24	0.24–0.35
**25) When I’m upset with someone, I tend to try to put myself in their place for a while. (PT)**	**0.49–0.60**	-0.14--0.07
**26) When a film is interesting, I wonder how I would feel if the events in the story were happening to me. (FS)**	**0.68–0.76**	−0.12--0.06
27) When I see someone who needs help in an emergency, I become torn apart. (PD)	0.23–0.37	0.20–0.30
**28) Before criticizing somebody, I try to imagine how I would feel if I were in their place. (PT)**	**0.46–0.60**	−0.27--0.17

Cognitive Empathy Subscales: Fantasy Scale (FS) and Perspective Taking (PT)

Affective Empathy Subscales: Personal Distress (PD) and Empathic Concern (EC)

“-” indicates that the question was originally negatively coded

Boldface indicates loadings greater than 0.40 over all 10 iterations or an item in the final scale

Italics indicates loadings with ranges that cross 0.40 over all 10 iterations

Final Cognitive Scale Questions: 5, 16, 21, 23, 25, 26, 28

Final Affective Scale Questions: 6, 10, 13, 14, 17, 18

The first factor contained ten items that crossed the 0.40 factor loading threshold in at least two of the ten multiply imputed datasets. Seven of those ten items were from cognitive subscales (fantasy and perspective taking). This seven-item subscale had an intra-scale Cronbach’s *α* of 0.78 and represents the cognitive empathy subscale (Table 3). The second factor contained nine items that crossed the 0.40 threshold in at least two of the imputed datasets. Six of those nine items were from affective subscales (personal distress and empathetic concern) with an intra-scale Cronbach’s *α* of 0.73. These six items represent the affective empathy subscale (Table 3).

**Table 3:** Intra-Scale Correlation for the Cognitive and Affective Scales

	Cognitive Subscale	*rho (95% CI)*	*p-value*	Affective Subscale	*rho (95% CI)*	*p-value*
Total *α*	0.78	Ref	Ref	0.73	0.621 (0.585, 0.655)	< 0.001*
Female *α*	0.78			0.74		
Male *α*	0.76			0.71		
PHQ-9 Score (0–27)		0.152 (0.093, 0.211)	< 0.0001*		0.075 (0.015, 0.135)	0.014*

Cognitive Scale Questions: 5, 16, 21, 23, 25, 26, 28

Affective Scale Questions: 6, 10, 13, 14, 17, 18

*95% CI* 95% confidence interval, *PHQ-9* Patient Health Questionaire-9

* indicates statistical significance at *α* = 0.05

### Confirmatory factor analysis and measurement invariance

Following the two-factor solution found by the EFA, we sought to confirm this factor structure within partner dyads using dyadic CFA and invariance testing. We did this by imposing gradually increased levels of measurement invariance (Table 4). Results were aggregated across ten multiply imputed data sets. First, we found that the configural model (*n* = 396 dyads, 792 individuals) – where the constellation of items and latent construct was compared for male vs female partners – offered the following fit to the data: CFI = 0.903, TLI = 0.887, RMSEA = 0.059. Second, we found that the model constrained to metric invariance had a similar, but marginally (and unexpectedly, because more constrained models generally are not a better fit to the data) *better* fit than the baseline configural model (CFI = 0.914, TLI = 0.904, RMSEA = 0.056, ΔCFI = 0.011 > 0.002). Lastly, we found that the scalar model provided a satisfactory fit, but did not fit the data as well as the metric model and did not exhibit scalar invariance (CFI = 0.897, TLI = 0.903, RMSEA = 0.057, ΔCFI = 0.017 > 0.002) [46]. Therefore, we determined that the two-factor model exhibited metric, but not scalar, invariance. Factor correlations ranged from 0.03 to 0.31 across imputed datasets.

**Table 4:** Adjusted and Unadjusted Fit Indices for Dyadic Invariance Testing Confirmatory Factor Analysis

	Configural Invariance Model		Metric Invariance Model		Scalar Invariance Model	
	Adjusted (Robust)	Unadjusted	Adjusted (Robust)	Unadjusted	Adjusted (Robust)	Unadjusted
Comparative Fit Index	0.903	0.939	0.914	0.953	0.897	0.941
Tucker Lewis Index	0.887	0.929	0.904	0.947	0.903	0.944
Root Mean Square Error of Approximation	0.059	0.062	0.056	0.061	0.057	0.063

After EFA, application of the underlying IRI theoretical framework, and dyadic CFA, our finalized empathy instrument included 13 of the 28 original IRI items. The cognitive and affective subscales suggested by EFA and supported by dyadic CFA were moderately correlated within each participant (Pearson’s rho: 0.621, 95% CI: 0.585, 0.655) (Table 3).

### Demographic comparisons

The exploratory and dyadic confirmatory factor analyses suggested a two-factor solution. To further probe the construct validity of this two-factor IRI model, we examined demographic and psychological differences on this adapted measure. These comparisons are confounded by the inability to establish scalar invariance (i.e., men and women interpreted the scaling of the items differently). Any statistically significant sex differences should therefore be interpreted with caution. We found unexpectedly lower mean scores on the cognitive subscale among women (Mean difference 95% CI: − 0.205, − 0.026) and those with a PHQ-9 score of 9 or lower (not depressed) at baseline (Mean difference 95% CI: − 0.352, − 0.120) and higher mean cognitive empathy score in participants 26 or younger (Mean difference 95% CI: 0.010, 0.196) (Table 5). We observed lower affective empathy scores among females (Mean difference 95% CI: − 0.186, − 0.0004) and those with more than 7 years of education (Mean difference 95% CI: − 0.247, − 0.024) and higher affective empathy scores in participants 26 or younger (Mean difference 95% CI: 0.010, 0.200) (Table 5). There is at least one district and one language that has a mean affective and cognitive empathy score that is statistically different from the others (Table 5). There was a low positive correlation (rho = 0.152, 95% CI: 0.093–0.211) between continuous PHQ-9 scores and cognitive empathy (Table 3).

**Table 5:** Demographic Comparisons of the Cognitive and Affective Scales

	Cognitive Scale (0–4)	*Mean Difference* *95% CI*	*p-value*	Affective Scale (0–4)	*Mean Difference* *95% CI*	*p-value*
Female (*n* = 595)	2.205	−0.205, − 0.026	0.012*^t^	2.368	− 0.186, − 0.0004	0.049*^t^
Male (*n* = 590)	2.320			2.461		
Age ≤ 26 (*n* = 693)	2.304	0.010, 0.196	0.031*^t^	2.457	0.010, 0.200	0.030*^t^
Age > 26 (*n* = 491)	2.201			2.353		
≤ 7 years of Education (*n* = 885)	2.246	−0.183, 0.037	0.191^t^	2.381	−0.247, − 0.024	0.017*^t^
> 7 years of Education (*n* = 204)	2.319			2.517		
No Depression (*n* = 894)	2.209	− 0.352, − 0.120	< 0.001*^t^	2.400	−0.200, 0.069	0.341^t^
Depression (*n* = 172)	2.444			2.465		
District			< 0.001*^a^			< 0.001*^a^
Pebane (*n* = 355)	2.316			2.454		
Inhassunge (*n* = 196)	2.277			2.386		
Gilé (*n* = 134)	2.233			2.270		
Quelimane (*n* = 21)	2.733			3.057		
Mocubela (*n* = 156)	2.601			2.666		
Namacurra (*n* = 229)	1.925			2.271		
Maganja da Costa (*n* = 94)	2.221			2.321		
Predicted Language			< 0.001*^a^			0.001*^a^
Muniga (*n* = 511)	2.403			2.519		
Chuabo (*n* = 446)	2.118			2.359		
Lomue (n = 134)	2.233			2.270		
Nharringa (n = 94)	2.221			2.321		

*95% CI* 95% confidence interval in an unpaired t-test

* indicates statistical significance at *α* = 0.05

t indicates *p*-value from an unpaired t-test

^a^ indicates *p*-value from a one-way analysis of variance (ANOVA)

## Discussion

Our exploratory factor analysis, consideration of the distinct cognitive and affective domains of empathy, and dyadic CFA suggested that a refined, shortened IRI scale is an acceptable measure of empathy in this population of adult partners living with HIV in Zambézia Province, Mozambique. Instead of the original four factor structure, exploratory factor analyses suggested, and the CFA with dyadic invariance testing confirmed, two empathy subscales: an affective subscale (with items assessing personal distress and empathetic concern) and a cognitive scale (with items assessing perspective taking and fantasy), which aligns with more recently developed empathy scales and recent data that describe the neurological processes underlying empathy [25–29]. The obtained subscales were moderately correlated with each other and had comparable intra-scale reliabilities among women and men.

This analysis takes an important initial step toward advancing our capacity to assess empathy changes associated with behavioral interventions in SSA, building upon insights from one province in Mozambique. This paper assesses empathy within romantic partners via a dyadic approach to more accurately capture the interdependence of this relational process. In turn, this yields a more precise assessment of partner empathy, which augments our capacity to assess how the HoPS+ intervention (in this case) or other couples-based interventions may change partner empathy, which itself may contribute to positive “downstream” treatment outcomes. For example, more empathic partners may better enable adherence to medication protocols, attending clinic care, and emotional support. This may also lead to interventions that target this important mediator of health intervention fidelity [13–17].

Our analysis established metric invariance, suggesting that the IRI items loaded similarly for male and female partners within the dyadic CFA analytic framework. Despite having a satisfactory fit, scalar invariance could not be established because of a statistically significant decreased fit from the metric invariance model (ΔCFI = 0.017 > 0.002). While the CFI and TLI fall below the commonly targeted thresholds of model fit proposed by Hu & Bentler [49], we feel the use of robust adjusted estimates, adoption of conservative ΔAFI thresholds, translation into four new languages, and application to an Mozambican sample with a median 5 years of formal education (as opposed to college-educated college-aged students) makes this fit a satisfactory introduction of this scale to a new population. More work can and should be done to further refine the measurement of cognitive and affective empathy in SSA.

Although our two-factor IRI had higher inter-scale correlations (0.62) within each participant than previous IRI or IRI for couple validations (range of 0.01–0.44), we had very similar intra-scale correlations and the dyadic CFA showed inter-scale correlations of 0.03–0.31 among partners [11, 19, 20, 30–32]. We cannot rule out that the higher intra-scale correlations are due to the new context for IRI questions.

The lack of scalar invariance across sex suggests that partners may exhibit different baseline values, limiting the degree to which mean differences (t-tests) can be interpreted across partners. In practice, this implies that partners with a latent empathy value of zero should not be expected to have the same average response on survey items. Thus, results from sex comparisons of empathy levels (e.g., men were observed to have higher levels of affective and cognitive empathy) ought to be considered with this in mind. Furthermore, similar empathy across both sexes aligns with our experience working with the communities in Zambézia Province. Higher empathy among younger individuals and higher empathy among those with more education aligns with previous research [55]. In contrast to a review that suggests higher affective empathy with depression – secondary to an increased focus on the self during a depressed state [58], we found no meaningful differences in affective empathy among patients with and without depression. However, our sample size is more than double that of all the reviewed studies combined and our participants are members of the community instead of mixed (hospitalized, on medications, etc.) and therefore are unlikely to act in the same way as those previously described [58].

Our study has several other limitations. Due to translation discrepancies, we excluded two items from the original IRI prior to starting our analyses. We excluded additional items when they loaded on the “incorrect” subscale (cognitive items on affective and vice versa), however, we feel it was reasonable to apply substantive considerations in this way, given the differences between this population and the populations on which the IRI was originally validated. Furthermore, having a more succinct scale will reduce missing responses when this scale is used in other settings. Additionally, about 11% of our participants were excluded from the analyses because they had more than two missing items per subscale. This resulted in a reduction of our sample size and statistical differences between the included and excluded participants.

Finally, the inclusion of four language groups in seven study districts resulted in substantial variability without a sufficient sample to conduct language- and/or district-specific analyses. Though we would have liked to have accounted for dyads within a language- or a district-nested model, the relatively low number of strata and sample sizes within some strata prevented these multilevel analyses. As our data collection continues, this may be a possibility for future research.

## Conclusions

In conclusion, our findings provide support for a two-subscale version of the IRI that measures cognitive and affective empathy among HIV-positive adults living in Zambézia Province, Mozambique. Given the association between empathy and health intervention fidelity, this new scale should be useful in assessing the effectiveness of interventions designed to increase social support among couples, family, and groups. This scale validation will help us measure the effect of the HoPS+ intervention on male and female empathy within the context of partner dyads, which, in turn, is hypothesized to impact adherence to treatment, retention in care, and maternal-to-child HIV transmission. Furthermore, this dyadic approach provides inroads to assess how constructs such as empathy, stigma, physician trust, and social support (other constructs measured in HoPS+) are related to each other and positive treatment outcomes for PLWH. Future applications and validations will allow health researchers to develop interventions that target empathy within partner dyads as a mediator of health intervention uptake in HIV and other chronic health conditions. Subsequent scale validations/adaptations would benefit from testing this scale in different languages and in different regions in Mozambique and/or SSA.

## Supplementary information

**Additional file 1 Supplemental Table 1.** Original and Adapted Interpersonal Reactivity Index (IRI).

## Data Availability

The datasets used and/or analyzed during the current study are available from the corresponding author on reasonable request.

## Abbreviations

PLWH: People living with HIV
SSA: Sub-Saharan Africa
HoPS+: Homens para Saúde Mais
IRI: Interpersonal Reactivity Index
PHQ-9: Patient Health Questionaire-9
EFA: Exploratory Factor Analysis
CFA: Confirmatory Factor Analysis
ΔAFI: Changes in alternative fit indices
ΔCFI: Changes in the confirmatory fit index
RMSEA: Root Mean Squared Error of Approximation
CFI: Comparative Fit Index
TLI: Tucker Lewis Index

## References

[1] (UNAIDS) JUNPoHA (2018). Fact Sheet – July 2018.

[2] IaM I (2017). Inquérito de Indicadores de Imunização, Malária e HIV/SIDA em Moçambique (IMASIDA 2015).

[3] John GC, Nduati RW, Mbori-Ngacha DA, Richardson BA, Panteleeff D, Mwatha A (2001). Correlates of mother-to-child human immunodeficiency virus type 1 (HIV-1) transmission: association with maternal plasma HIV-1 RNA load, genital HIV-1 DNA shedding, and breast infections. J Infect Dis.

[4] Audet CM, Blevins M, Chire YM, Aliyu MH, Vaz LM, Antonio E (2016). Engagement of men in antenatal care services: increased HIV testing and treatment uptake in a community participatory action program in Mozambique. AIDS Behav.

[5] Audet CM, Graves E, Barreto E, De Schacht C, Gong W, Shepherd BE (2018). Partners-based HIV treatment for seroconcordant couples attending antenatal and postnatal care in rural Mozambique: A cluster randomized trial protocol. Contemp Clin Trials.

[6] Audet CM, Graves E, Bravo M, Aliyu MH, Alvim F, Green AF (2017). Male engagement strategies effective in improving Option B+ retention in rural Mozambique.

[7] Dunlap J, Foderingham N, Bussell S, Wester CW, Audet CM, Aliyu MH (2014). Male involvement for the prevention of mother-to-child HIV transmission: A brief review of initiatives in east, west, and Central Africa. Curr HIV/AIDS Rep.

[8] Farquhar C, Kiarie JN, Richardson BA, Kabura MN, John FN, Nduati RW (2004). Antenatal couple counseling increases uptake of interventions to prevent HIV-1 transmission. J Acquir Immune Defic Syndr (1999).

[9] Ghanotakis E, Hoke T, Wilcher R, Field S, Mercer S, Bobrow EA (2017). Evaluation of a male engagement intervention to transform gender norms and improve family planning and HIV service uptake in Kabale, Uganda. Global Public Health.

[10] Peltzer K, Jones D, Weiss SM, Shikwane E (2011). Promoting male involvement to improve PMTCT uptake and reduce antenatal HIV infection: a cluster randomized controlled trial protocol. BMC Public Health.

[11] Davis MH. A multidimensional approach to individual differences in empathy. J Pers Soc Psychol. 1980;10:85.

[12] Dvash J, Shamay-Tsoory SG (2014). Theory of mind and empathy as multidimensional constructs neurological foundations. Top Lang Disord.

[13] Leiberg S, Anders S (2006). The multiple facets of empathy: a survey of theory and evidence. Prog Brain Res.

[14] Derksen F, Bensing J, Lagro-Janssen A (2013). Effectiveness of empathy in general practice: a systematic review. Br J Gen Pract.

[15] Flickinger TE, Saha S, Roter D, Korthuis PT, Sharp V, Cohn J (2016). Clinician empathy is associated with differences in patient-clinician communication behaviors and higher medication self-efficacy in HIV care. Patient Educ Couns.

[16] Mercer SW, Higgins M, Bikker AM, Fitzpatrick B, McConnachie A, Lloyd SM (2016). General practitioners' empathy and health outcomes: A prospective observational study of consultations in areas of high and low deprivation. Ann Fam Med.

[17] Lin C, Li L, Wan D, Wu Z, Yan Z (2012). Empathy and avoidance in treating patients living with HIV/AIDS (PLWHA) among service providers in China. AIDS Care.

[18] Devoldre I, Davis MH, Verhofstadt LL, Buysse A (2010). Empathy and social support provision in couples: social support and the need to study the underlying processes. J Psychol..

[19] Peloquin K, Lafontaine MF (2010). Measuring empathy in couples: validity and reliability of the interpersonal reactivity index for couples. J Pers Assess.

[20] Levesque C, Lafontaine MF, Carona A, Flesch JL, Bjornson S (2014). Dyadic Empathy, Dyadic Coping, and Relationship Satisfaction: A Dyadic Model. Europe’s J Psychologty.

[21] Verhofstadt L, Devoldre I, Buysse A, Stevens M, Hinnekens C, Ickes W (2016). The role of cognitive and affective empathy in Spouses' support interactions: an observational study. PLoS One.

[22] Harris PA, Taylor R, Thielke R, Payne J, Gonzalez N, Conde JG (2009). Research Electronic Data Capture (REDCap)--a metadata-driven methodology and workflow process for providing translational research informatics support. J Biomed Inform.

[23] USAID (2019). Education Washington, DC.

[24] The World Bank (2018). Mozambique Economic Update: Less Poverty, but More Inequality.

[25] Innamorati M, Ebisch SJH, Gallese V, Saggino A (2019). A bidimensional measure of empathy: empathic experience scale. PLoS One.

[26] Batchelder L, Brosnan M, Ashwin C (2017). The development and validation of the empathy components questionnaire (ECQ). PLoS One.

[27] Cox CL, Uddin LQ, Di Martino A, Castellanos FX, Milham MP, Kelly C (2012). The balance between feeling and knowing: affective and cognitive empathy are reflected in the brain's intrinsic functional dynamics. Soc Cogn Affect Neurosci.

[28] Reniers RL, Corcoran R, Drake R, Shryane NM, Vollm BA (2011). The QCAE: a questionnaire of cognitive and affective empathy. J Pers Assess.

[29] Shamay-Tsoory SG (2011). The neural bases for empathy. Neuroscientist..

[30] De Corte K, Buysse A, Verhofstadt LL, Roeyers H, Ponnet K, Davis MH (2007). Measuring empathic tendencies: reliability and validity of the Dutch version of the interpersonal reactivity index. Psychol Belg.

[31] Fernandez AM, Dufey M, Kramp U (2011). Testing the psychometric properties of the interpersonal reactivity index (IRI) in Chile empathy in a different cultural context. Eur J Psychol Assess.

[32] Siu AMH, Shek DTL (2005). Validation of the interpersonal reactivity index in a Chinese context. Res Social Work Prac.

[33] American Psychiatric Association (2013). DSM-5 Task Force. Diagnostic and statistical manual of mental disorders : DSM-5.

[34] Cholera R, Gaynes BN, Pence BW, Bassett J, Qangule N, Macphail C (2014). Validity of the patient health questionnaire-9 to screen for depression in a high-HIV burden primary healthcare clinic in Johannesburg, South Africa. J Affect Disord.

[35] Pence BW, Gaynes BN, Atashili J, O'Donnell JK, Tayong G, Kats D (2012). Validity of an interviewer-administered patient health questionnaire-9 to screen for depression in HIV-infected patients in Cameroon. J Affect Disord.

[36] Monahan PO, Shacham E, Reece M, Kroenke K, Ong'or WO, Omollo O (2009). Validity/reliability of PHQ-9 and PHQ-2 depression scales among adults living with HIV/AIDS in Western Kenya. J Gen Intern Med.

[37] Audet CM, Wainberg ML, Oquendo MA, Yu QR, Peratikos MB, Duarte CS (2018). Depression among female heads-of-household in rural Mozambique: A cross-sectional population-based survey. J Affect Disord.

[38] van Buuren S, Groothuis-Oudshoorn K (2011). Mice: multivariate imputation by chained equations in R. J Stat Softw.

[39] Barnfather N, Amod Z (2012). Empathy and personal experiences of trainees in an emotional literacy and persona doll programme in South Africa. S Afr J Psychol.

[40] MacRitchie V, Leibowitz S (2010). Secondary traumatic stress, level of exposure, empathy and social support in trauma workers. S Afr J Psychol.

[41] Team RC (2018). R: A language and environment for statistical computing.

[42] Henson RK, Roberts JK (2006). Use of exploratory factor analysis in published research - common errors and some comment on improved practice. Educ Psychol Meas.

[43] Costello AB, Osborne J. Best Practices in Exploratory Factor Analysis: Four Recommendations for Getting the Most From Your Analysis. Pract Assess Res Eval. 2005;10:7.

[44] Cook WL, Kenny DA (2005). The actor-partner interdependence model: A model of bidirectional effects in developmental studies. Int J Behav Dev.

[45] Rosseel Y (2012). Lavaan: an R package for structural equation modeling. J Stat Softw.

[46] Meade AW, Johnson EC, Braddy PW (2008). Power and sensitivity of alternative fit indices in tests of measurement invariance. J Appl Psychol.

[47] Brosseau-Liard PE, Savalei V (2014). Adjusting incremental fit indices for nonnormality. Multivar Behav Res.

[48] Brosseau-Liard PE, Savalei V, Li LB (2012). An investigation of the sample performance of two nonnormality corrections for RMSEA. Multivar Behav Res.

[49] Hu L-t, Bentler PM (1999). Cutoff criteria for fit indexes in covariance structure analysis: conventional criteria versus new alternatives. Struct Equ Model.

[50] Little TD (2013). Longitudinal structural equation modeling.

[51] Putnick DL, Bornstein MH (2016). Measurement invariance conventions and reporting: the state of the art and future directions for psychological research. Dev Rev.

[52] Sakaluk JK, Kilshaw R, Fisher AN, Leshner CE. Dyadic measurement invariance and its importance for replicability in romantic relationship research. preprint. PsyArXiv; 2019 2019/03/06/.

[53] Chen FF (2007). Sensitivity of goodness of fit indexes to lack of measurement invariance. Struct Equ Model Multidiscip J.

[54] Satorra A, Bentler PM (1994). Corrections to test statistics and standard errors in covariance structure analysis. Latent variables analysis: Applications for developmental research.

[55] Gruhn D, Rebucal K, Diehl M, Lumley M, Labouvie-Vief G (2008). Empathy across the adult lifespan: longitudinal and experience-sampling findings. Emotion..

[56] Levy J, Goldstein A, Feldman R (2019). The neural development of empathy is sensitive to caregiving and early trauma. Nat Commun.

[57] O'Connor LE, Berry JW, Weiss J, Gilbert P (2002). Guilt, fear, submission, and empathy in depression. J Affect Disord.

[58] Schreiter S, Pijnenborg GHM, Aan Het Rot M (2013). Empathy in adults with clinical or subclinical depressive symptoms. J Affect Disord.

[59] Thoma P, Zalewski I, von Reventlow HG, Norra C, Juckel G, Daum I (2011). Cognitive and affective empathy in depression linked to executive control. Psychiatry Res.

[60] Tully EC, Ames AM, Garcia SE, Donohue MR (2016). Quadratic associations between empathy and depression as moderated by emotion dysregulation. J Psychol.

