# Supplementary material for: Interpersonal reactivity index adaptation among expectant seroconcordant couples with HIV in Zambézia Province, Mozambique
Source: BMC Psychol. 2020 Aug 28;8:90. doi: 10.1186/s40359-020-00442-0 (PMC7456002; doi:10.1186/s40359-020-00442-0)
Supplement: Supplementary file 1 — Additional file 1 Supplemental Table 1. Original and Adapted Interpersonal Reactivity Index (IRI). [file 40359_2020_442_MOESM1_ESM.docx]

| **Supplemental Table 1: Original and Adapted Interpersonal Reactivity Index (IRI)** | | |
| --- | --- | --- |
| Question (subscale) | Original Questions (Davis 1980) | Adapted Questions* |
| 1 (FS) | I daydream and fantasize, with some regularity, about things that might happen to me. | I imagine and dream, with some regularity, about things that might happen to me. |
| 2 (EC) | I often have tender, concerned feelings for people less fortunate than me. | I often have feelings of affection and concern for people less happy than me. |
| 3- (PT) | I sometimes find it difficult to see things from the "other guy's" point of view. | I can see things from "another person’s" point of view. |
| 4- (EC) | Sometimes I don't feel sorry for other people when they are having problems. | I do feel sorry for other people when I have problems. |
| **5 (FS)** | **I really get involved with the feelings of the characters in a novel.** | **I really get involved with the feelings of the characters in a movie.** |
| **6 (PD)** | **In emergency situations, I feel apprehensive and ill-at-ease.** | **In emergency situations, I feel afraid and ill- disposed.** |
| 7- (FS) | I am usually objective when I watch a movie or play, and I don't often get completely  caught up in it. | I'm not normally objective when I watch a movie or game, and I often get completely  caught up in it. |
| 8 (PT) | I try to look at everybody's side of a disagreement before I make a decision. | I try to look at everybody's side of a disagreement before I make a decision. |
| 9 (EC) | When I see someone being taken advantage of, I feel kind of protective toward them. | When I see someone taken advantage of, I feel a little protective against them. |
| **10 (PD)** | **I sometimes feel helpless when I am in the middle of a very emotional situation.** | **Sometimes I feel helpless when I am in the midst of a very emotional situation.** |
| 11 (PT) | I sometimes try to understand my friends better by imagining how things look from their perspective. | Sometimes, to try to understand my friends better, I imagine how things seem from their perspective. |
| 12- (FS) | Becoming extremely involved in a good book or movie is somewhat rare for me. | It's a common for me to become heavily involved in a good book or movie. |
| **13- (PD)** | **When I see someone get hurt, I tend to remain calm.** | **When I see someone get hurt, I usually don’t stay calm.** |
| **14- (EC)** | **Other people's misfortunes do not usually disturb me a great deal.** | **The misfortunes of other people usually disturb me much.** |
| 15- (PT) | If I'm sure I'm right about something, I don't waste much time listening to other people's arguments. | Even if I'm sure I'm right about something, I spend time listening to other people's arguments. |
| **16 (FS)** | **After seeing a play or movie, I have felt as though I were one of the characters.** | **After seeing a play or movie, I feel like I'm one of the characters.** |
| **17 (PD)** | **Being in a tense emotional situation scares me.** | **Being in an emotional and tense situation scares me.** |
| **18- (EC)** | **When I see someone being treated unfairly, I sometimes don't feel very much pity for them.** | **When I see someone being treated unfairly, I feel much pity for them.** |
| 19- (PD) | I am usually pretty effective in dealing with emergencies. | I tend to be ineffective in dealing with emergencies. |
| 20 (EC) | I am often quite touched by things that I see happen. | I am often very touched by things that I see happen. |
| **21 (PT)** | **I believe that there are two sides to every question and try to look at them both.** | **I believe there are two sides to every question and I usually look at both.** |
| 22 (EC) | I would describe myself as a pretty soft-hearted person. | I would describe myself as a very kind person. |
| **23 (FS)** | **When I watch a good movie, I can very easily put myself in the place of a leading**  **character.** | **When I watch a good movie, I can easily put myself in the place of the main character.** |
| 24 (PD) | I tend to lose control during emergencies. | I tend to lose control during emergencies. |
| **25 (PT)** | **When I'm upset at someone, I usually try to "put myself in his shoes" for a while.** | **When I'm upset with someone, I tend to try to put myself in their place for a while.** |
| **26 (FS)** | **When I am reading an interesting story or novel, I imagine how I would feel if the events in the story were happening to me.** | **When a film is interesting, I wonder how I would feel if the events in the story were happening to me.** |
| 27 (PD) | When I see someone who badly needs help in an emergency, I go to pieces. | When I see someone who needs help in an emergency, I become torn apart. |
| **28 (PT)** | **Before criticizing somebody, I try to imagine how I would feel if I were in their place.** | **Before criticizing somebody, I try to imagine how I would feel if I were in their place.** |

Cognitive Empathy Subscales: Fantasy Scale (FS) and Perspective Taking (PT)

Affective Empathy Subscales: Personal Distress (PD) and Empathic Concern (EC)

“-“ indicates that the question was originally negatively coded

* Homens para Saúde Mais (HoPS+) trial questions after translation to Portuguese and adaptation to the cultural norms in Zambézia Province, Mozambique

Bolded items are in the final scales

Final Cognitive Scale Questions: 5, 16, 21, 23, 25, 26, 28

Final Affective Scale Questions: 6, 10, 13, 14, 17, 18

**Sample Size Justification:**

*Exploratory Factor Analysis*: we assessed the loadings of 26 items (after excluding two items for the full 28-item Interpersonal Reactivity Index *a priori* due to inappropriate translations) in 343 individuals (after excluding 57 individuals with excessive missing data). This means that we included 13 individuals per item. From 26 items, we narrowed our item pool to 13 items and two factors, 6.5 items per factor. Finally, we used loadings of greater than 0.40 (over 10 imputations) to select items for moving to the next step in the analysis (comparisons with previous Interpersonal Reactivity Index validations and other empathy scales). We followed Costello and Osborne’s (2005) recommendations to maximize sample size per item ratio and our loading threshold for the most reliable results (42).

*Dyadic Confirmatory Factor Analysis (CFA)*: Our dyadic CFA consisted of invariance testing carried out on 466 unique dyads. Previous Monte Carlo simulation studies have shown that goodness of fit indices in the context of dyadic data can vary in their consistency based on sample size. This research has made recommendations for sample sizes according to these findings (50, 51, 60). Minimal sample sizes should be at least 200 unique dyads, whereas 400 unique dyads are described as adequate (50, 51, 60). Additionally, Sakaluk (2019) describes sample sizes above 400 as “well above the norm” for typical recommendations needed for invariance testing (50). In dividing the sample for use in the EFA and CFA, we used these results to guide our distribution.
